# Physical activity, smoking, and genetic predisposition to obesity in people from Pakistan: the PROMIS study

**DOI:** 10.1186/s12881-015-0259-x

**Published:** 2015-12-18

**Authors:** Shafqat Ahmad, Wei Zhao, Frida Renström, Asif Rasheed, Maria Samuel, Mozzam Zaidi, Nabi Shah, Nadeem Hayyat Mallick, Khan Shah Zaman, Mohammad Ishaq, Syed Zahed Rasheed, Fazal-ur-Rheman Memon, Bashir Hanif, Muhammad Shakir Lakhani, Faisal Ahmed, Shahana Urooj Kazmi, Philippe Frossard, Paul W. Franks, Danish Saleheen

**Affiliations:** Genetic and Molecular Epidemiology Unit, Lund University Diabetes Centre, Department of Clinical Sciences, Lund University, Malmö, Sweden; Perelman School of Medicine at the University of Pennsylvania, Philadelphia, PA USA; Center for Non-Communicable Diseases Pakistan, Karachi, Pakistan; Department of Pharmacy, COMSATS Institute of Information Technology, Abbottabad, Pakistan; Punjab Institute of Cardiology, Lahore, Pakistan; National Institute of Cardiovascular Diseases, Karachi, Pakistan; Karachi Institute of Heart Diseases, Karachi, Pakistan; Red Crescent Institute of Cardiology, Hyderabad, Karachi Pakistan; Tabba Heart Institute, Karachi, Pakistan; Department of Cardiology, Liaquat National Hospital, Karachi, Pakistan; Department of Microbiology, University of Karachi, Karachi, Pakistan; Nazarbayev University, Astana, Kazakhstan; Department of Public Health and Clinical Medicine, Section for Medicine, Umeå University, Umeå, Sweden; Department of Nutrition, Harvard School of Public Health, Boston, MA USA; Department of Biostatistics and Epidemiology, University of Pennsylvania, Philadelphia, PA USA

**Keywords:** Obesity, Physical activity, Smoking, Genetic susceptibility, Gene-lifestyle interactions

## Abstract

**Background:**

Multiple genetic variants have been reliably associated with obesity-related traits in Europeans, but little is known about their associations and interactions with lifestyle factors in South Asians.

**Methods:**

In 16,157 Pakistani adults (8232 controls; 7925 diagnosed with myocardial infarction [MI]) enrolled in the PROMIS Study, we tested whether: a) BMI-associated loci, individually or in aggregate (as a genetic risk score - GRS), are associated with BMI; b) physical activity and smoking modify the association of these loci with BMI. Analyses were adjusted for age, age^2^, sex, MI (yes/no), and population substructure.

**Results:**

Of 95 SNPs studied here, 73 showed directionally consistent effects on BMI as reported in Europeans. Each additional BMI-raising allele of the GRS was associated with 0.04 (SE = 0.01) kg/m^2^ higher BMI (*P* = 4.5 × 10^−14^). We observed nominal evidence of interactions of *CLIP1* rs11583200 (*P*_interaction_ = 0.014), *CADM2* rs13078960 (*P*_interaction_ = 0.037) and *GALNT10* rs7715256 (*P*_interaction_ = 0.048) with physical activity, and *PTBP2* rs11165643 (*P*_interaction_ = 0.045), *HIP1* rs1167827 (*P*_interaction_ = 0.015), *C6orf106* rs205262 (*P*_interaction_ = 0.032) and *GRID1* rs7899106 (*P*_interaction_ = 0.043) with smoking on BMI.

**Conclusions:**

Most BMI-associated loci have directionally consistent effects on BMI in Pakistanis and Europeans. There were suggestive interactions of established BMI-related SNPs with smoking or physical activity.

**Electronic supplementary material:**

The online version of this article (doi:10.1186/s12881-015-0259-x) contains supplementary material, which is available to authorized users.

## Background

Obesity and its numerous metabolic, atherogenic, osteoarthritic, and metastatic comorbidities [1–4] place enormous burdens on health care systems, societies and individuals worldwide. The global prevalence of obesity has increased substantially in recent decades; in 2010, it was estimated that overweight and obesity caused 3·4 million deaths worldwide [5]. Obesity and its comorbidities present major health and financial challenges in South Asia, with Pakistan being the ninth highest-ranking country in terms of the global burden of obesity [5]. As in many other societies, the obesity epidemic in South Asians is driven by the recent, widespread adoption of Westernized lifestyles causing chronic positive energy balance [6]. In South Asians, however, it is widely believed that these changes in lifestyle are set against a genetic background that renders this population especially susceptible to the adverse cardiometabolic consequences of obesity.

While the specific genetic aberrations that give rise to the high heritability estimates observed for obesity have been studied extensively in Europeans [7], few studies have been reported in indigenous South Asian populations. The identities of 97 independent loci that harbor BMI–associated variants are now established in European–ancestry populations [8]. Other studies of European-ancestry populations have explored whether some of these loci interact with physical activity and smoking to affect obesity predisposition [9–15], but none has been reported in indigenous South Asians to date.

This study was undertaken in 16,157 ethnic Pakistani adults from the Pakistan Risk of Myocardial Infarction Study (PROMIS). The aim of the study was to examine genetic associations and gene-lifestyle interactions for BMI-associated variants previously identified and replicated in European-ancestry populations [8]. We focused on comparing the direction and magnitude of the genetic association signals between European and Pakistani adults; we also sought to determine if smoking or physical activity modified these effects.

## Methods

### Study sample

PROMIS is a case–control study of acute myocardial infarction (MI) in participants recruited from six centres in urban Pakistan. Frequency-matched controls by age (5-year strata) and sex were identified from patients attending the outpatient clinic for routine checkups, and visitors to the hospital (including non-blood related visitors of PROMIS cases). Non-fasting blood samples were collected from each participant. For MI cases data were collected within 24 h of the onset of symptoms. A detailed description of the PROMIS study, including participant selection criteria, has been published elsewhere [16–18]. The participants were enrolled from the National Institute of Cardiovascular Disorders Karachi, Karachi Institute of Institute of Heart Diseases Karachi, Red Crescent Hospital Hyderabad, Punjab Institute of Cardiology Lahore, Multan Institute of Cardiology Multan, and Faisalabad Institute of Cardiology, Faisalabad. All participants provided written informed consent and the study was approved by the research ethics committee of the Center for Non-Communicable Diseases (CNCD) Pakistan and also by regional Ethical Review committees in the different centres across Pakistan involved in the study. In-addition to the institutional review board (IRB) at CNCD, Karachi, IRBs at National Institute of Cardiovascular Disorders, Karachi, Punjab Institute of Cardiology, Lahore, and Tabba Heart Institute, Karachi approved the study.

### Body composition and exposure assessment

Height and weight were measured using calibrated wall-mounted stadiometers and balance-beam scales, respectively. BMI was calculated as weight in kilograms (kg) divided by height in meters squared (m^2^). Lifestyle factors including smoking and physical activity were assessed using validated questionnaires administered by trained research medical officers [16]. We defined <18.5 kg/m^2^ as ‘underweight’; 18.5–22.9 kg/m^2^ as ‘normal weight’; 23–27.5 kg/m^2^ as ‘overweight’; and >27.5 kg/m^2^ as ‘obese’, concordant with the WHO recommendations for Asian populations [19].

To quantify physical activity and tobacco exposure, we first developed a pilot questionnaire. For exposure to tobacco consumption, with the help of local dietician and physicians, we came up with list of all tobacco items that are typically consumed in the Pakistani population. Similarly, for physical activity, with the help of an exercise physiologist, questions pertaining to level of activity at work, at home, mode of transportation used for commuting to work (e.g., bicycle, walking), nature of activities engaged at leisure time and nature of job were assessed in the pilot questionnaire. The pilot questionnaire was further used to assess the mode and frequency of tobacco consumption and nature of physical activity in 300 participants who were randomly chosen from an urban resident population in Karachi. The pilot questionnaire also sought information on any other forms of tobacco consumption or physical activity through open ended questions to capture information not covered by the pilot questionnaire. Based on the responses received from the participants, the questionnaire was finalized. Exposure to tobacco consumption was divided into: “ever” or “never” or “current” and information on “smoked”, “chewable”, or “snuffed” forms of tobacco was separately sought. For physical activity, participants were categorized to have “low”, “moderate”, or “intense” physical activity based on their responses. We recognize that we have not used any objective measures to quantify “physical activity” or “exposure to tobacco”; however the magnitude of the inverse association between our estimates of physical activity and smoking with BMI in PROMIS is comparable to what has been reported elsewhere for validated instruments, strongly supporting the validity of our measures. The physical activity variable was constructed by the cross-tabulation of occupational, leisure time, and commuting physical activity, such that the variable categorizes a person’s total physical activity into three levels: (i) light (ii) moderate and (iii) heavy. Participants were categorized into never-smokers, ex-smokers and current-smokers [17] and for the current analyses; a binary smoking variable was created by merging the categories for ‘ex-smokers’ and ‘never-smokers’ (as smoking cessation is likely to convey different effects on BMI than current smoking). Sensitivity analyses were also conducted in current smokers vs never smokers by excluding ex-smokers participants and the genetic estimates were not materially different (results not shown).

### Genotyping

DNA was extracted from peripheral blood leukocytes using a phenolchloroform protocol [17]. Genotyping was performed using the Illumina Human 650 K/ Illumina Human OmniExpress 770 K. In order to minimize potential bias attributable to plate- or batch-specific genotyping errors, DNA plates contained a mixture of cases and controls, including blank samples [18]. SNPs with departure from Hardy-Weinberg equilibrium (*P*-value = 1.0 × 10^−6^), call rate < 95 % or minor allele frequency <1 % were excluded from the analyses. Participants who were cryptically related or those with ambiguous reported sex or with a missing rate for genetic data >5 % were also dropped from the analyses. The GWAS data was subsequently imputed to the global 1000 genomes reference panel (March 2012 (v3)).

Two of the 97 BMI-associated genetic variants (*NRXN3* rs10150332 and *SLC39A8* rs1310732) [8], did not pass genotyping quality control, and so were excluded from the present analyses. Genotype information and quality control of the remaining 95 BMI-associated variants are shown in Additional file 1: Table S1 and allele frequencies were consistent with those reported for GIH (Gujarati Indians in Houston, Texas) population. For the 26 SNPs not reported for GIH, allele frequencies were compared with CEU (Utah residents with Northern and Western European ancestry from the CEPH collection) (http://www.ncbi.nlm.nih.gov/variation/tools/1000genomes) in 1000 Genome (Additional file 2: Table S4). Hardy-Weinberg equilibrium for 95 BMI associated SNPs were calculated in control samples as shown in Additional file 1: Table S1.

### Statistical analyses

An un-weighted genetic risk score (GRS) was calculated based upon 95 BMI-associated variants by summing the number of BMI-associated alleles [8]. We chose not to weight the GRS, as the large databases from where those weightings might be obtained are comprised predominantly of European–ancestry populations, which we felt might bias the comparison of our findings with previous studies in Europeans. All variables (e.g., age, BMI and GRS) were normally distributed. General linear models (GLM) were used to assess the association of genotypes (individual SNPs or GRS) with BMI assuming an additive effect. All the models were adjusted for age, age^2^, sex, case/control status and population substructure (first ten genetic principle components; PCs). Logistic regression was used to calculate odds ratios (ORs) for the association of the GRS with obesity (BMI > 27.5 vs 18.5 ≤ BMI < 23). To quantify the descriminative ability of these 95 BMI-associated variants, the area under the receiver operating characteristic curve (ROC AUC) was generated from the logistic regression model and compared using roccomp package in STATA. Generally, it is not advisable to study gene-lifestyle interactions in diseased people, especially when the lifestyle exposures of interest are commonly known risk factors for the disease; therefore we undertook the explicit tests of gene-lifestyle interactions in non-diseased PROMIS participants only. Although the main effect analyses of SNP/GRS were performed in all participants, including cases, doing so is not susceptible to response bias, as this type of analyses does not involve the use of self-reported lifestyle data. Indeed, the inclusion of these additional 7925 cases in the main effect analysis may be advantageous, as it likely increases statistical power and ensures a degree of independence in the populations studied. Interaction analyses for individual SNPs and GRS were performed by additionally introducing the product term (SNP/GRS x lifestyle exposure) along with the marginal effect terms in the model. All analyses were performed using SAS version 9.4 (SAS Institute, Cary, NC) and STATA (version 12, Stata Corp, College Station, TX, USA) and the analysis script is available from the authors on request.

## Results

Participant characteristics are shown in Table 1. In total, 16,157 individuals (7925 MI cases/8232 controls) had baseline data available for these analyses. More men (82 %) than women (18 %) participated in this study. The GRS has a mean value of 87 risk alleles and minimum and maximum values of 64 and 112, respectively.

**Table 1:** Characteristics of the PROMIS study participants

Trait	Total sample (*N* = 16,157)	Controls (*N* = 8232)	Cases (*N* = 7925)
Sex (male/female) %	82/18	80/20	84/16
BMI (kg/m^2^)	25.8 ± 4.1	25.9 ± 4.3	25.8 ± 3.9
Age (years)	53.8 ± 9.6	54.1 ± 8.9	53.6 ± 10.3
Genetic risk score	87 ± 6	87 ± 6	87 ± 6
^a^Smoking – N (%)			
Never	8045 (50)	4810 (58)	3235 (41)
Ex-smokers	1495 (9)	819 (10)	676 (9)
Current	6537 (41)	2590 (32)	3497 (50)
Physical activity- N (%)			
Light	5261 (39)	2749 (41)	2512 (38)
Moderate	6788 (51)	3401 (50)	3387 (51)
Heavy	1394 (10)	634 (9)	760 (11)

Data are means ± SD unless otherwise indicated

^a^Among controls, *N* = 13 and *N* = 1448 participants miss lifestyle data on smoking and physical activity, respectively

### Main effect of SNP/GRS on BMI

**Fig. 1:**
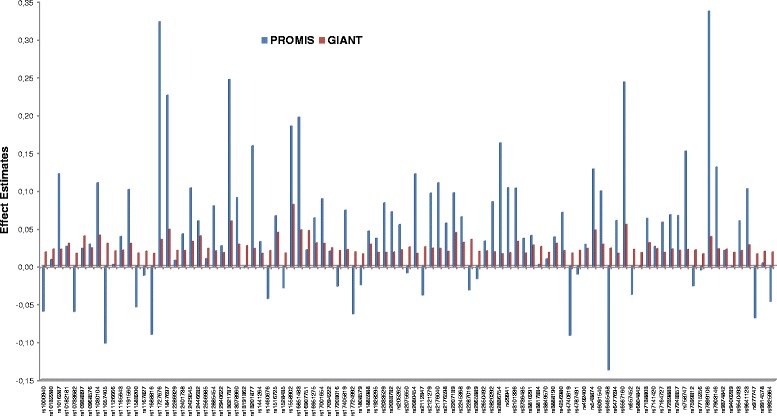
Effect estimates for the 95 BMI variants obtained in the PROMIS study (*N* = 16,157) in comparison to those reported by the GIANT Consortium (Locke et al. [8]) (*N* = 339,224)

Of the 95 BMI-susceptibility SNPs, 73 showed directionally consistent associations with BMI as reported in the original study [8]; of which 22 loci (*FLJ30838, BDNF, EHBP1, HHIP, PRKD1, TMEM18, FTO, RASA2, CBLN1, LOC646736, ETS2, KCTD15, NEGR1, C9orf93, SEC16B, ZFP64, ASB4, MC4R, NLRC3, GRID1, TCF7L2* and *CALCR*) reached *P* < 0.05 (Fig. 1, Table 2). Consistent with previous studies in European populations, the most strongly BMI-associated variant (lowest *p*-value) in Pakistanis was *MC4R* rs6567160, β = 0.24 (SE = 0.05) kg/m^2^ per copy of the C allele; (*P* = 1.2 × 10^−7^) (Table 2). In total, these 95 BMI associated variants explain 1.54 % of the phenotypic variance in BMI in the PROMIS cohort, which is less than the 2.7 % reported in Locke et al. [8].

**Fig. 2:**
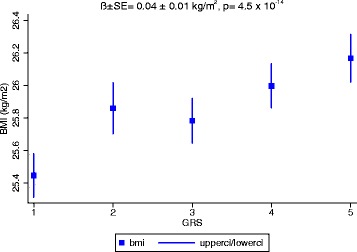
Association of the genetic risk score (GRS) based on 95 BMI-associated SNPs and BMI in the PROMIS Study (*N* = 16,157). Data represents mean (95 % CI) BMI per quintile of the GRS, adjusted forage, age^2^, sex, MI status, and principal components

**Table 2:** Cross-sectional association of 95 BMI associated SNPs on BMI in the total PROMIS cohort (*N* = 16,157)

SNP	Nearest gene	Effect/other allele	β (kg/m^2^/allele)	SE	*P*
rs1000940	*RABEP1*	G/A	−0.06	0.05	0.19
rs10132280	*STXBP6*	C/A	0.01	0.06	0.87
rs1016287	*FLJ30838*	T/C	0.12	0.05	0.02
rs10182181	*ADCY3*	G/A	0.03	0.04	0.55
rs10733682	*LMX1B*	A/G	−0.06	0.04	0.18
rs10938397	*GNPDA2*	G/A	0.02	0.05	0.59
rs10968576	*LINGO2*	G/A	0.03	0.05	0.58
rs11030104	*BDNF*	A/G	0.11	0.05	0.03
rs11057405	*CLIP1*	G/A	−0.10	0.15	0.48
rs11126666	*KCNK3*	A/G	0.00	0.05	0.95
rs11165643	*PTBP2*	T/C	0.04	0.04	0.37
rs11191560	*NT5C2*	C/T	0.10	0.06	0.07
rs11583200	*ELAVL4*	C/T	−0.05	0.04	0.23
rs1167827	*HIP1*	G/A	−0.01	0.04	0.79
rs11688816	*EHBP1*	G/A	−0.09	0.04	0.04
rs11727676	*HHIP*	T/C	0.32	0.15	0.03
rs11847697	*PRKD1*	T/C	0.23	0.08	0.01
rs12286929	*CADM1*	G/A	0.01	0.05	0.85
rs12401738	*FUBP1*	A/G	0.04	0.05	0.42
rs12429545	*OLFM4*	A/G	0.10	0.06	0.07
rs12446632	*GPRC5B*	G/A	0.06	0.10	0.56
rs12566985	*FPGT-TNNI3K*	G/A	0.01	0.05	0.82
rs12885454	*PRKD1*	C/A	0.08	0.05	0.08
rs12940622	*RPTOR*	G/A	0.03	0.05	0.57
rs13021737	*TMEM18*	G/A	0.25	0.06	3.15E-05
rs13078960	*CADM2*	G/T	0.09	0.07	0.20
rs13191362	*PARK2*	A/G	0.00	0.07	0.98
rs13201877	*IFNGR1*	G/A	0.16	0.08	0.05
rs1441264	*MIR548A2*	A/G	0.03	0.05	0.50
rs1460676	*FIGN*	C/T	−0.04	0.06	0.49
rs1516725	*ETV5*	C/T	0.07	0.06	0.27
rs1528435	*UBE2E3*	T/C	−0.03	0.05	0.54
rs1558902	*FTO*	A/T	0.19	0.05	8.85E-05
rs16851483	*RASA2*	T/G	0.20	0.07	0.01
rs16907751	*ZBTB10*	C/T	0.02	0.06	0.69
rs16951275	*MAP2K5*	T/C	0.06	0.05	0.17
rs17001654	*SCARB2*	G/C	0.09	0.08	0.25
rs17094222	*HIF1AN*	C/T	0.02	0.06	0.75
rs17203016	*CREB1*	G/A	−0.03	0.06	0.67
rs17405819	*HNF4G*	T/C	0.07	0.05	0.12
rs17724992	*PGPEP1*	A/G	−0.06	0.04	0.16
rs1808579	*C18orf8*	C/T	−0.02	0.04	0.58
rs1885988	*MTIF3*	C/T	0.05	0.08	0.54
rs1928295	*TLR4*	T/C	0.04	0.05	0.41
rs2033529	*TDRG1*	G/A	0.08	0.06	0.14
rs2033732	*RALYL*	C/T	0.07	0.05	0.16
rs205262	*C6orf106*	G/A	0.06	0.05	0.31
rs2075650	*TOMM40*	A/G	−0.01	0.07	0.90
rs2080454	*CBLN1*	C/A	0.12	0.05	0.02
rs2112347	*POC5*	T/G	−0.04	0.04	0.39
rs2121279	*LRP1B*	T/C	0.10	0.10	0.32
rs2176040	*LOC646736*	A/G	0.11	0.05	0.03
rs2176598	*HSD17B12*	T/C	0.06	0.06	0.31
rs2207139	*TFAP2B*	G/A	0.10	0.05	0.07
rs2245368	*PMS2L11*	C/T	0.07	0.05	0.16
rs2287019	*QPCTL*	C/T	−0.03	0.06	0.61
rs2365389	*FHIT*	C/T	−0.02	0.04	0.71
rs2650492	*SBK1*	A/G	0.03	0.06	0.56
rs2820292	*NAV1*	C/A	0.09	0.04	0.06
rs2836754	*ETS2*	C/T	0.16	0.05	7.45E-04
rs29941	*KCTD15*	G/A	0.10	0.05	0.02
rs3101336	*NEGR1*	C/T	0.10	0.05	0.03
rs3736485	*DMXL2*	A/G	0.04	0.04	0.41
rs3810291	*ZC3H4*	A/G	0.04	0.04	0.36
rs3817334	*MTCH2*	T/C	0.00	0.05	0.95
rs3849570	*GBE1*	A/C	0.01	0.05	0.83
rs3888190	*ATP2A1*	A/C	0.04	0.05	0.45
rs4256980	*TRIM66*	G/C	0.07	0.04	0.11
rs4740619	*C9orf93*	T/C	−0.09	0.04	0.04
rs4787491	*INO80E*	G/A	−0.01	0.04	0.81
rs492400	*USP37*	C/T	0.03	0.05	0.54
rs543874	*SEC16B*	G/A	0.13	0.06	0.04
rs6091540	*ZFP64*	C/T	0.10	0.05	0.04
rs6465468	*ASB4*	T/G	−0.14	0.06	0.02
rs6477694	*EPB41L4B*	C/T	0.06	0.04	0.18
rs6567160	*MC4R*	C/T	0.24	0.05	1.15E-07
rs657452	*AGBL4*	A/G	−0.04	0.04	0.40
rs6804842	*RARB*	G/A	0.00	0.04	0.98
rs7138803	*BCDIN3D*	A/G	0.06	0.05	0.16
rs7141420	*NRXN3*	T/C	0.03	0.04	0.56
rs7164727	*LOC100287559*	T/C	0.06	0.05	0.20
rs7239883	*LOC284260*	G/A	0.07	0.05	0.17
rs7243357	*GRP*	T/G	0.07	0.05	0.21
rs758747	*NLRC3*	T/C	0.15	0.05	1.29E-03
rs7599312	*ERBB4*	G/A	−0.03	0.06	0.66
rs7715256	*GALNT10*	G/T	0.00	0.04	0.91
rs7899106	*GRID1*	G/A	0.34	0.14	0.01
rs7903146	*TCF7L2*	C/T	0.13	0.05	0.01
rs9374842	*LOC285762*	T/C	0.02	0.05	0.65
rs9400239	*FOXO3*	C/T	0.00	0.04	1.00
rs9540493	*MIR548X2*	A/G	0.06	0.04	0.17
rs9641123	*CALCR*	C/G	0.10	0.05	0.02
rs977747	*TAL1*	T/G	−0.07	0.04	0.13
rs9914578	*SMG6*	G/C	0.00	0.05	0.92
rs9925964	*KAT8*	A/G	−0.05	0.06	0.41

Analyses were adjusted for age, age^2^, sex, MI status, genetic principal components (first 10)

In the overall sample, each unit increase (equivalent to one effect allele) in the GRS was positively associated with BMI (β = 0.04 (SE = 0.01) kg/m^2^ per allele; *P* = 4.5 × 10^−14^) as shown in (Fig. 2). When the GRS was categorized into quintiles, the mean BMI difference between the highest and lowest quintile was 0.54 kg/m^2^ (Fig. 1). Each additional GRS risk allele conveyed an odds ratio (OR) for obesity of 1.02 (95 % CI: 1.02, 1.03; *P* = 1.2 × 10^−10^). The association of each SNP and the GRS with BMI were directionally consistent in control and case samples, and there was no evidence of effect modification by disease state (results not shown).

### Discriminative accuracy

**Fig. 3:**
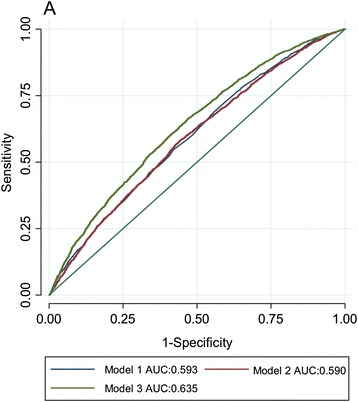
The discriminative power of different models to predict risk of obesity [BMI (kg/m^2^) ≥27.5 compared with 18.5 kg/m^2^ < BMI < 23 kg/m^2^]. The area under the receiver operating characteristic curve (ROC AUC) is investigated: Model 1 = age, age^2^, sex and case status; model 2 = all 95 single nucleotide polymorphisms; model 3 = model 1 + model 2

The discriminative accuracy (ROC AUC) for obesity [obese (*N* = 5079); BMI ≥27.5 kg/m^2^ compared with normal weight (*N* = 3512); 18.5 kg/m^2^ ≤ BMI < 23 kg/m^2^] of the model including only the 95 SNPs was 0.590 (95 % CI: 0.549, 0.573) and the discriminative accuracy of the model including only age, age^2^, sex, case/control status was 0.593 (95 % CI: 0.581, 0.606); adding genotypes to the latter model increased (*P* <0.0001) the AUC to 0.635 (95 % CI: 0.623, 0.647) (Fig. 3).

### Gene-lifestyle interactions

**Fig. 4:**
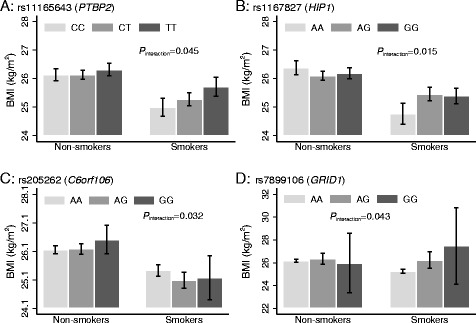
Association of *PTBP2* rs11165643 (**a**), *HIP1* rs1167827 (**b**), *C6orf106* rs205262 (**c**) and *GRID1* rs7899106 (**d**), on BMI stratified by smoking status in control participants (*N* = 8219) from PROMIS

We tested each of the 95 BMI-associated SNPs for interactions with physical activity and smoking on BMI in the control sample only. None of these results remained statistically significant after correction for multiple testing (*P* < 0.00026 = *P* = 0.05/190 tests). We observed nominally significant SNP x smoking interactions for *PTBP2* rs11165643 (*P*_interaction_ = 0.045), *HIP1* rs1167827 (*P*_interaction_ = 0.015), *C6orf106* rs205262 (*P*_interaction_ = 0.032) and *GRID1* rs7899106 (*P*_interaction_ = 0.043) (Fig. 4). In the case of *PTBP2* rs11165643, the association of the T-allele on BMI was larger among smokers (β = 0.36 kg/m^2^/per allele, SE = 0.12, *P*-value = 0.002) compared to non-smokers (β = 0.09 kg/m^2^/per allele, SE = 0.08, *P*-value = 0.26). A similar trend of larger effect among smokers compared to non-smokers was observed for *HIP1* rs1167827 and *GRID1* rs7899106 variants. Whereas, the effect of the G-allele at *C6orf106* rs205262 on BMI was higher among non-smokers compared to smokers (Additional file 3: Table S2).

**Fig. 5:**
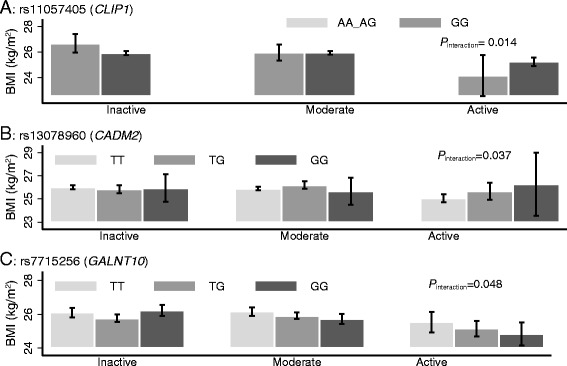
Association between *CLIP1* rs11057405 (**a**) *CADM2* rs13078960 (**b**) and *GALNT10* rs7715256 (**c**) on BMI stratified by physical activity in control participants (*N* = 6784) from PROMIS. For *CLIP1* rs11057405 variant, there were only *N* = 7 participants are in the “active” physical activity group, so participants having genotype “AA” and “AG” were combined for the Fig. 5a

We also observed suggestive evidence of interactions between physical activity and the *CLIP1* rs11057405 (*P*_interaction_ = 0.014), *CADM2* rs13078960 (*P*_interaction_ = 0.037) and *GALNT10* rs7715256 (*P*_interaction_ = 0.048) loci on BMI (Fig. 5 and Additional file 4: Table S3). There was no evidence of an interaction between the GRS and physical activity or smoking on BMI.

## Discussion

We examined genetic associations and gene-lifestyle interactions for 95 established obesity-susceptibility variants in relation to BMI in 16,157 Pakistani adults. To our knowledge, this is the first study examining genetic associations and gene-lifestyle interactions for established obesity-susceptibility loci in an indigenous Pakistani population, and the largest and most comprehensive study of gene-lifestyle interactions conducted in non-European populations to date.

The associations of most (77 %) obesity-susceptibility variants with BMI, although not all statistically significant, were directionally consistent with findings from European-ancestry populations (Fig. 1). We also observed comparable allele frequencies in our population with those reported in the HapMap GIH (Gujarati Indians in Houston, USA) population. The GIH is a population emanating from the western Indian state of Gujarat who migrated to Houston, Texas. The distribution of the allele frequencies of most of the variants (83 out of 95) in this Pakistani population was also comparable to that reported in populations of European origin [8], reflecting similar linkage disequilibrium patterns in the Pakistani and European genomes.

The *MC4R* (rs6567160) and *TMEM18* (rs13021737) loci showed the strongest associations (lowest *P*-values) with BMI (0.24 and 0.25 kg/m^2^ higher BMI per risk allele with *P* = 1.1 × 10^−07^ and *P* = 3.1 × 10^−05^, respectively) in this study. We observed nominal interactions with smoking for the *PTBP2* rs11165643, *HIP1* rs1167827, *C6orf106* rs205262 and *GRID1* rs7899106 on BMI. We also observed nominal interactions of physical activity with *CLIP1* rs11583200, *CADM2* rs13078960 and *GALNT10* rs7715256 on BMI. None of these interaction effects remained statistically significant after Bonferroni correction for multiple testing; hence these results should be viewed purely as hypothesis generating and ones that will benefit from further scrutiny in independent cohorts and settings. Although in previous studies of European-ancestry populations, it has been reported that physical activity can attenuate the genetic predisposition conveyed by *FTO* variants [9–13], no such interactions were evident within the PROMIS cohort, which suggests that the observations reported elsewhere might be population-specific or attributable to confounding factors that are absent in this indigenous Pakistani cohort.

*CLIP1* encodes a protein called CAP-GLY domain containing linker protein 1, which secures endocytic vesicles to microtubules, playing a potentially important role in atherosclerosis via LDL transportation and in a range of cancers including Hodgkin’s lymphoma and anaplastic large cell lymphoma [20]. *CLIP1* is also a target of the cellular energy-sensing enzyme AMPK [21, 22], but no studies have been published to our knowledge relating this locus to energy homeostasis, exercise, smoking or diet. Cell adhesion molecules 2 (*CADM2*) gene which is also called *Necl-3*, *IGSF4D* or *SynCAM 2*, involves in cell aggregation and organization of functional synapses through heterophilic adhesion [23]. It has been shown that *CADM2* is expressed in nervous system of zebra fish [24], implicating that the *CADM2* is a conserved evolutionarily gene and may be implicated in various physiological processes related to obesity in humans. *GALNT10* (polypeptide N-acetylgalactosaminyltransferase 10) are post-translational modification of secreted and membrane-associated proteins and has important role in normal development of cellular processes [25]. *PTBP2* (polypyrimidine tract binding protein 2) is an intensely studied RNA binding protein involved in several post -transcriptional regulatory events of gene expression including exon splicing. High levels of expression of *PTBP2* gene is observed in adult brain and muscles which implicate its role in the physiology of cardiometabolic phenotypes [26]. Huntingtin interacting protein 1 (*HIP1*) is a component of clathrin coats which is involved in the binding to membrane phospholipids and these properties contribute to their ability to stimulate clathrin assembly at specific sites on the plasma membrane [27].

A strong cumulative effect of the 95 variants on obesity was also noted, with each additional risk allele corresponding to approximately 0.04 kg/m^2^ units, or 115 g for a person 1.70 m tall, which under a non-epistatic additive genetic model amounts to an estimated difference in weight of ~4.2 kg for persons 1.70 m tall at the 75 % vs 25 % percentile of the risk allele distribution. Combined, the 95 SNPs explained 1.54 % of the phenotypic variance in BMI suggests that many common variants having small effects, with the vast majority of the heritable variance yet to be explained [7, 28].

Consistent with the findings that these SNPs explain a small proportion of variation in BMI, the 95 SNPs combined had litle discriminative power (the ROC AUC for the model including only these 95 variants was 0.590), although adding these 95 SNPs to a model including age, age^2^, sex and case/control status did significantly (*p* < 0.0001) improve the discriminative ability of the model (Fig. 3). Li et al. [29] studied 12 of the 95 BMI susceptibility variants examined here and reported similar findings. Sandholt et al. [30] reported that the ROC AUC for 20 BMI-associated SNPs for the risk of overweight and obesity was 0.53 and 0.58. These results suggests that currently available genetic information for obesity have little discriminative ability and are too weak for clinical utility.

Our study is limited by the use of self-reported lifestyle data, which was obtained in cases soon after an MI had occurred; in principle, self-reported data for lifestyle habits that are known in the Pakistani population to be associated with cardiovascular disease (such as smoking), may be prone to bias. To address this limitation, we performed analyses stratified by case and control cohorts, as well as in both combined; however, results were largely consistent between groups, suggesting that bias of this nature is unlikely to have impacted our results to a meaningful degree (results not shown). A further important limitation of this study is that with many statistical comparisons performed some of our nominally significant associations are likely to be false positive. However, because many of our analyses are replication tests, for which a Bonferroni correction is likely to be overly conservative, we elected to present the number of tests performed alongside their nominal *P*-values so that the reader can determine for themselves the value of these findings.

## Conclusions

In conclusion, most common genetic variants for BMI identified through GWAS in Europeans have small and directionally consistent effects on obesity risk in this Pakistani population, with the *MC4R* and *TEMEM18* loci conveying the largest effects. Smoking and physical activity may modify the genetic predisposition to obesity at numerous loci in Pakistanis, but replication and extension of these findings into other South Asian populations is needed.

## Availability of data and materials

“The raw data are not publicly available owing to the study’s data access policies. However, analyses in the PROMIS dataset can be requested by contacting the corresponding author”.

## Additional files

Additional file 1: Table S1. Quality control descriptive of 95 BMI associated SNPs in the PROMIS Cohort (*N* = 16,157). (DOCX 52 kb) Additional file 2: Table S4. Quality control information for the included SNPs (95 published BMI-associated) that were directly genotyped and also the imputed data of the SNPs for which imputation was performed in the PROMIS Cohort (*N* = 16,157). (DOCX 22 kb) Additional file 3: Table S2. Associations of the 95 SNPs on BMI by different strata of smoking (non-smokers vs current smokers) in control participants from PROMIS (*N* = 8219). (DOCX 43 kb) Additional file 4: Table S3. Association of individual 95 SNPs with different strata of physical activity on BMI in control (*N* = 6784) participants from PROMIS. (DOCX 53 kb)
