# Supplementary material for: Physical activity, smoking, and genetic predisposition to obesity in people from Pakistan: the PROMIS study
Source: BMC Med Genet. 2015 Dec 18;16:114. doi: 10.1186/s12881-015-0259-x (PMC4683724; doi:10.1186/s12881-015-0259-x)
Supplement: Additional file 1: Table S1. — Quality control descriptive of 95 BMI associated SNPs in the PROMIS Cohort (N = 16,157). (DOCX 52 kb) [file 12881_2015_259_MOESM1_ESM.docx]

**Table S1**: Quality control descriptive of 95 BMI associated SNPs in the PROMIS Cohort (N= 16,157).

| **SNP** | **Chr, Position** | **Nearest Gene** | **Effect/other allele** | **EAF** | **EAF (GIH)** | **EAF GIANT** | **HWE** |
| --- | --- | --- | --- | --- | --- | --- | --- |
| rs1000940 | 17:5,223,976 | *RABEP1* | G/A | 0.42 | 0.37 | 0.32 | 0.86 |
| rs10132280 | 14:24,998,019 | *STXBP6* | C/A | 0.80 | 0.73 | 0.68 | 0.03 |
| rs1016287 | 2:59,159,129 | *FLJ30838* | T/C | 0.24 | 0.25 | 0.29 | 0.04 |
| rs10182181 | 2:25,003,800 | *ADCY3* | G/A | 0.47 | 0.42 | 0.46 | 0.60 |
| rs10733682 | 9:128,500,735 | *LMX1B* | A/G | 0.42 | 0.49 | 0.48 | 0.11 |
| rs10938397 | 4:45,182,527 | *GNPDA2* | G/A | 0.41 | 0.44 | 0.43 | 0.14 |
| rs10968576 | 9:28,404,339 | *LINGO2* | G/A | 0.21 | 0.13 | 0.32 | 0.08 |
| rs11030104 | 11:27,641,093 | *BDNF* | A/G | 0.75 | 0.75 | 0.79 | 3.24E-04 |
| rs11057405 | 12:121,347,850 | *CLIP1* | G/A | 0.98 | 0.97 | 0.90 | 0.22 |
| rs11126666 | 2:26,782,315 | *KCNK3* | A/G | 0.30 | 0.25 | 0.28 | 3.31E-03 |
| rs11165643 | 1:96,696,685 | *PTBP2* | T/C | 0.49 | 0.43 | 0.58 | 3.86E-04 |
| rs11191560 | 10:104,859,028 | *NT5C2* | C/T | 0.19 | 0.25 | 0.09 | 0.01 |
| rs11583200 | 1:50,332,407 | *ELAVL4* | C/T | 0.51 | 0.60 | 0.40 | 0.15 |
| rs1167827 | 7:75,001,105 | *HIP1* | G/A | 0.56 | 0.61 | 0.55 | 2.52E-03 |
| rs11688816 | 2:62,906,552 | *EHBP1* | G/A | 0.57 | 0.63 | 0.53 | 0.01 |
| rs11727676 | 4:145,878,514 | *HHIP* | T/C | 0.98 | 0.97 | 0.91 | 0.29 |
| rs11847697 | 14:30,515, 112 | *PRKD1* | T/C | 0.07 | 0.02 | 0.04 | 0.43 |
| rs12286929 | 11:114,527,614 | *CADM1* | G/A | 0.42 | 0.41 | 0.52 | 0.24 |
| rs12401738 | 1:78,219,349 | *FUBP1* | A/G | 0.21 | 0.18 | 0.35 | 0.02 |
| rs12429545 | 13:53,000,207 | *OLFM4* | A/G | 0.18 | 0.14 | 0.13 | 0.09 |
| rs12446632 | 16:19,842,890 | *GPRC5B* | G/A | 0.95 | 0.96 | 0.87 | 2.12E-03 |
| rs12566985 | 1:75,002,193 | *FPGT-TNNI3K* | G/A | 0.40 | 0.48 | 0.45 | 0.39 |
| rs12885454 | 14:28,806,589 | *PRKD1* | C/A | 0.63 | 0.63 | 0.64 | 1.06E-04 |
| rs12940622 | 17:76,230,166 | *RPTOR* | G/A | 0.69 | 0.75 | 0.58 | 1.97E-06 |
| rs13021737 | 2:62,23;48 | *TMEM18* | G/A | 0.83 | 0.87 | 0.83 | 0.01 |
| rs13078960 | 3:85,807,590 | *CADM2* | G/T | 0.11 | 0.22 | 0.20 | 0.01 |
| rs13191362 | 6:162,953,340 | *PARK2* | A/G | 0.90 | 0.94 | 0.88 | 3.36E-03 |
| rs13201877 | 6:137,675,541 | *IFNGR1* | G/A | 0.08 | 0.11 | 0.14 | 0.01 |
| rs1441264 | 13:78,478,920 | *MIR548A2* | A/G | 0.71 | 0.73 | 0.61 | 0.03 |
| rs1460676 | 2:164,275,935 | *FIGN* | C/T | 0.15 | 0.13 | 0.18 | 2.52E-03 |
| rs1516725 | 3:185,824,004 | *ETV5* | C/T | 0.84 | 0.87 | 0.87 | 1.36E-04 |
| rs1528435 | 2:181,259,207 | *UBE2E3* | T/C | 0.66 | 0.70 | 0.63 | 0.02 |
| rs1558902 | 16:53,803,574 | *FTO* | A/T | 0.34 | 0.45 | 0.42 | 0.05 |
| rs16851483 | 3:142,758,126 | *RASA2* | T/G | 0.11 | 0.11 | 0.07 | 0.02 |
| rs16907751 | 8:81,375,457 | *ZBTB10* | C/T | 0.81 | 0.90 | 0.91 | 0.05 |
| rs16951275 | 15:68,077,168 | *MAP2K5* | T/C | 0.66 | 0.79 | 0.78 | 0.03 |
| rs17001654 | 4:77,348,592 | *SCARB2* | G/C | 0.09 | 0.06 | 0.15 | 0.21 |
| rs17094222 | 10:102,385,430 | *HIF1AN* | C/T | 0.14 | 0.12 | 0.21 | 1.99E-03 |
| rs17203016 | 2:207,963,763 | *CREB1* | G/A | 0.15 | 0.10 | 0.20 | 0.09 |
| rs17405819 | 8:76,806,584 | *HNF4G* | T/C | 0.67 | 0.72 | 0.70 | 0.50 |
| rs17724992 | 19:18,315,825 | *PGPEP1* | A/G | 0.44 | 0.49 | 0.75 | 0.03 |
| rs1808579 | 18:19,358,886 | *C18orf8* | C/T | 0.48 | 0.47 | 0.53 | 0.09 |
| rs1885988 | 13:26,915,782 | MTIF3 | C/T | 0.09 | 0.11 | 0.20 | 0.01 |
| rs1928295 | 9:119,418,304 | *TLR4* | T/C | 0.61 | 0.61 | 0.55 | 0.15 |
| rs2033529 | 6:40,456,631 | *TDRG1* | G/A | 0.19 | 0.15 | 0.29 | 0.09 |
| rs2033732 | 8:85,079,720 | *RALYL* | C/T | 0.75 | 0.77 | 0.74 | 3.34E-03 |
| rs205262 | 6:34,671,142 | *C6orf106* | G/A | 0.22 | 0.15 | 0.27 | 0.95 |
| rs2075650 | 19:50,087,459 | *TOMM40* | A/G | 0.89 | 0.88 | 0.85 | 0.14 |
| rs2080454 | 16:47,620,091 | *CBLN1* | C/A | 0.26 | 0.22 | 0.41 | 0.02 |
| rs2112347 | 5:75,015,242 | *POC5* | T/G | 0.53 | 0.63 | 0.63 | 2.00E-03 |
| rs2121279 | 2:142,759,755 | *LRP1B* | T/C | 0.05 | 0.05 | 0.15 | 4.60E-03 |
| rs2176040 | 2:227,092,802 | *LOC646736* | A/G | 0.25 | 0.30 | 0.37 | 0.03 |
| rs2176598 | 11:43,864,278 | *HSD17B12* | T/C | 0.18 | 0.28 | 0.25 | 8.45E-06 |
| rs2207139 | 6:50,845,490 | *TFAP2B* | G/A | 0.22 | 0.18 | 0.18 | 0.06 |
| rs2245368 | 7:76,446,079 | *PMS2L11* | C/T | 0.36 | 0.50 | 0.18 | 0.01 |
| rs2287019 | 19:50,894,012 | *QPCTL* | C/T | 0.85 | 0.82 | 0.80 | 2.34E-03 |
| rs2365389 | 3:61,236,462 | *FHIT* | C/T | 0.54 | 0.69 | 0.58 | 1.56E-03 |
| rs2650492 | 16:28,240,912 | *SBK1* | A/G | 0.18 | 0.25 | 0.30 | 0.01 |
| rs2820292 | 1:200,050,910 | *NAV1* | C/A | 0.56 | 0.62 | 0.56 | 0.05 |
| rs2836754 | 21:39,213,610 | *ETS2* | C/T | 0.30 | 0.26 | 0.56 | 2.38E-04 |
| rs29941 | 19:39,001,372 | *KCTD15* | G/A | 0.63 | 0.69 | 0.67 | 0.14 |
| rs3101336 | 1:72,523,773 | *NEGR1* | C/T | 0.66 | 0.59 | 0.61 | 0.22 |
| rs3736485 | 15:49,535,902 | *DMXL2* | A/G | 0.42 | 0.49 | 0.45 | 0.01 |
| rs3810291 | 19:52,260,843 | *ZC3H4* | A/G | 0.44 | 0.44 | 0.67 | 0.09 |
| rs3817334 | 11:47,607,569 | *MTCH2* | T/C | 0.30 | 0.29 | 0.41 | 0.01 |
| rs3849570 | 3:81,874,802 | *GBE1* | A/C | 0.33 | 0.39 | 0.36 | 1.01E-03 |
| rs3888190 | 16:28,796,987 | *ATP2A1* | A/C | 0.24 | 0.28 | 0.40 | 3.28E-03 |
| rs4256980 | 11:8,673,939 | *TRIM66* | G/C | 0.56 | 0.68 | 0.65 | 0.02 |
| rs4740619 | 9:15,624,326 | *C9orf93* | T/C | 0.53 | 0.52 | 0.54 | 0.01 |
| rs4787491 | 16:29,922,838 | *INO80E* | G/A | 0.46 | 0.50 | 0.51 | 0.55 |
| rs492400 | 2:219,057,996 | *USP37* | C/T | 0.33 | 0.32 | 0.42 | 0.20 |
| rs543874 | 1:176,156,103 | *SEC16B* | G/A | 0.15 | 0.19 | 0.19 | 0.01 |
| rs6091540 | 20:51,087,862 | *ZFP64* | C/T | 0.72 | 0.76 | 0.72 | 5.39E-04 |
| rs6465468 | 7:95,007,450 | *ASB4* | T/G | 0.18 | 0.20 | 0.31 | 7.55E-04 |
| rs6477694 | 9:110,972,163 | *EPB41L4B* | C/T | 0.55 | 0.65 | 0.37 | 0.21 |
| rs6567160 | 18:57,829,135 | *MC4R* | C/T | 0.38 | 0.25 | 0.24 | 0.08 |
| rs657452 | 1:49,362,434 | *AGBL4* | A/G | 0.50 | 0.58 | 0.39 | 0.23 |
| rs6804842 | 3:25,081,441 | *RARB* | G/A | 0.46 | 0.40 | 0.57 | 1.49E-03 |
| rs7138803 | 12:48,533,735 | *BCDIN3D* | A/G | 0.40 | 0.39 | 0.38 | 0.01 |
| rs7141420 | 14:78,969,207 | *NRXN3* | T/C | 0.57 | 0.58 | 0.53 | 0.02 |
| rs7164727 | 15:70,881,044 | *LOC100287559* | T/C | 0.62 | 0.60 | 0.67 | 1.86E-04 |
| rs7239883 | 18:38,401,669 | *LOC284260* | G/A | 0.27 | 0.22 | 0.39 | 3.03E-05 |
| rs7243357 | 18:56,883,319 | *GRP* | T/G | 0.77 | 0.84 | 0.81 | 4.33E-03 |
| rs758747 | 16:3,627,358 | *NLRC3* | T/C | 0.33 | 0.25 | 0.27 | 0.17 |
| rs7599312 | 2:213,121,476 | *ERBB4* | G/A | 0.82 | 0.88 | 0.72 | 1.96E-03 |
| rs7715256 | 5:153,518,086 | *GALNT10* | G/T | 0.45 | 0.48 | 0.42 | 7.45E-05 |
| rs7899106 | 10:87,400,884 | *GRID1* | G/A | 0.03 | 0.04 | 0.05 | 8.31E-06 |
| rs7903146 | 10:114,748,339 | *TCF7L2* | C/T | 0.69 | 0.72 | 0.71 | 0.01 |
| rs9374842 | 6:120,227,364 | *LOC285762* | T/C | 0.65 | 0.60 | 0.74 | 4.72E-04 |
| rs9400239 | 6:108,977,663 | *FOXO3* | C/T | 0.53 | 0.75 | 0.69 | 0.08 |
| rs9540493 | 13:65,103,705 | *MIR548X2* | A/G | 0.52 | 0.56 | 0.45 | 0.07 |
| rs9641123 | 7:93,197,732 | *CALCR* | C/G | 0.39 | 0.39 | 0.43 | 2.05E-04 |
| rs977747 | 1:47,457,264 | *TAL1* | T/G | 0.50 | 0.46 | 0.40 | 0.03 |
| rs9914578 | 17:1,951,886 | *SMG6* | G/C | 0.32 | 0.19 | 0.23 | 0.11 |
| rs9925964 | 16:31,129,895 | *KAT8* | A/G | 0.81 | 0.56 | 0.62 | 3.97E-04 |

EAF: Effect Allele Frequency * Allele frequency according to 1000-G CEU as data in GIH is unavailable
