# Supplementary material for: Physical activity, smoking, and genetic predisposition to obesity in people from Pakistan: the PROMIS study
Source: BMC Med Genet. 2015 Dec 18;16:114. doi: 10.1186/s12881-015-0259-x (PMC4683724; doi:10.1186/s12881-015-0259-x)
Supplement: Additional file 2: Table S4. — Quality control information for the included SNPs (95 published BMI-associated) that were directly genotyped and also the imputed data of the SNPs for which imputation was performed in the PROMIS Cohort (N = 16,157). (DOCX 22 kb) [file 12881_2015_259_MOESM2_ESM.docx]

Table S4 Quality control information for the included SNPs (95 published BMI-associated) that were directly genotyped and also the imputed data of the SNPs for which imputation was performed in the PROMIS Cohort (N= 16,157).

| **SNP** | **gwas1_info** | **gwas1_genotyped (Y/N)** | **gwas2_info** | **gwas2_genotyped (Y/N)** |
| --- | --- | --- | --- | --- |
| rs1000940 | 1 | Y | 0.995 | N |
| rs10132280 | 0.942 | N | 1 | Y |
| rs1016287 | 0.993 | N | 1 | Y |
| rs10182181 | 1 | Y | 0.977 | N |
| rs10733682 | 1 | Y | 1 | Y |
| rs10938397 | 0.995 | N | 1 | Y |
| rs10968576 | 1 | Y | 1 | Y |
| rs11030104 | 1 | Y | 1 | Y |
| rs11057405 | 1 | Y | 1 | Y |
| rs11126666 | 1 | Y | 1 | Y |
| rs11165643 | 1 | Y | 0.99 | N |
| rs11191560 | 0.982 | N | 1 | Y |
| rs11583200 | 1 | Y | 1 | Y |
| rs1167827 | 1 | Y | 1 | Y |
| rs11688816 | 1 | Y | 0.982 | N |
| rs11727676 | 0.68 | N | 1 | Y |
| rs11847697 | 0.957 | N | 0.969 | N |
| rs12286929 | 0.944 | N | 1 | Y |
| rs12401738 | 0.997 | N | 0.997 | N |
| rs12429545 | 1 | Y | 1 | Y |
| rs12446632 | 1 | Y | 0.999 | N |
| rs12566985 | 0.999 | N | 1 | N |
| rs12885454 | 0.976 | N | 0.987 | N |
| rs12940622 | 1 | Y | 1 | Y |
| rs13021737 | 0.91 | N | 0.996 | N |
| rs13078960 | 0.997 | N | 0.998 | N |
| rs13191362 | 0.957 | N | 0.947 | N |
| rs13201877 | 0.968 | N | 0.963 | N |
| rs1441264 | 1 | Y | 1 | Y |
| rs1460676 | 0.978 | N | 0.984 | N |
| rs1516725 | 0.997 | N | 0.998 | N |
| rs1528435 | 0.989 | N | 0.994 | N |
| rs1558902 | 0.997 | N | 1 | Y |
| rs16851483 | 0.994 | N | 1 | Y |
| rs16907751 | 0.956 | N | 0.981 | N |
| rs16951275 | 0.997 | N | 0.999 | N |
| rs17001654 | 0.861 | N | 0.841 | N |
| rs17094222 | 0.957 | N | 0.948 | N |
| rs17203016 | 1 | Y | 1 | Y |
| rs17405819 | 0.989 | N | 0.999 | N |
| rs17724992 | 1 | Y | 1 | Y |
| rs1808579 | 1 | Y | 0.962 | N |
| rs1885988 | 1 | Y | 0.947 | N |
| rs1928295 | 1 | Y | 1 | Y |
| rs2033529 | 1 | Y | 1 | Y |
| rs2033732 | 0.924 | N | 1 | Y |
| rs205262 | 1 | Y | 1 | Y |
| rs2075650 | 1 | Y | 1 | Y |
| rs2080454 | 0.983 | N | 1 | Y |
| rs2112347 | 0.98 | N | 0.985 | N |
| rs2121279 | 0.953 | N | 1 | Y |
| rs2176040 | 1 | N | 0.999 | N |
| rs2176598 | 0.936 | N | 1 | Y |
| rs2207139 | 0.999 | N | 0.999 | N |
| rs2245368 | 0.718 | N | 1 | Y |
| rs2287019 | 1 | Y | 1 | Y |
| rs2365389 | 0.985 | N | 0.992 | N |
| rs2650492 | 1 | Y | 0.923 | N |
| rs2820292 | 0.981 | N | 0.978 | N |
| rs2836754 | 1 | Y | 1 | Y |
| rs29941 | 1 | Y | 1 | Y |
| rs3101336 | 1 | Y | 1 | Y |
| rs3736485 | 0.939 | N | 1 | Y |
| rs3810291 | 0.941 | N | 1 | Y |
| rs3817334 | 0.99 | N | 1 | Y |
| rs3849570 | 0.994 | N | 0.997 | N |
| rs3888190 | 0.999 | N | 1 | Y |
| rs4256980 | 0.998 | N | 0.998 | N |
| rs4740619 | 1 | Y | 1 | Y |
| rs4787491 | 0.981 | N | 1 | Y |
| rs492400 | 0.987 | N | 1 | Y |
| rs543874 | 0.994 | N | 1 | Y |
| rs6091540 | 1 | N | 1 | N |
| rs6465468 | 0.938 | N | 0.931 | N |
| rs6477694 | 1 | Y | 1 | Y |
| rs6567160 | 0.995 | N | 1 | Y |
| rs657452 | 1 | Y | 1 | Y |
| rs6804842 | 1 | Y | 1 | Y |
| rs7138803 | 1 | Y | 1 | Y |
| rs7141420 | 1 | Y | 1 | Y |
| rs7164727 | 1 | Y | 0.962 | N |
| rs7239883 | 1 | Y | 1 | Y |
| rs7243357 | 0.978 | N | 1 | Y |
| rs758747 | 0.801 | N | 1 | Y |
| rs7599312 | 1 | Y | 1 | Y |
| rs7715256 | 0.981 | N | 0.996 | N |
| rs7899106 | 0.952 | N | 0.987 | N |
| rs7903146 | 1 | Y | 1 | Y |
| rs9374842 | 0.986 | N | 0.996 | N |
| rs9400239 | 1 | Y | 0.991 | N |
| rs9540493 | 0.938 | N | 1 | Y |
| rs9641123 | 0.979 | N | 0.96 | N |
| rs977747 | 0.994 | N | 1 | Y |
| rs9914578 | 0.996 | N | 1 | Y |
| rs9925964 | 1 | N | 1 | N |

Y=Yes imputed, N=No imputation

Two different genotyping platforms (gwas1_info and gwas2_info) in our study and an info-score for the imputed SNPs have been provided below for each of the platform used.
