# Supplementary material for: Physical activity, smoking, and genetic predisposition to obesity in people from Pakistan: the PROMIS study
Source: BMC Med Genet. 2015 Dec 18;16:114. doi: 10.1186/s12881-015-0259-x (PMC4683724; doi:10.1186/s12881-015-0259-x)
Supplement: Additional file 4: Table S3. — Association of individual 95 SNPs with different strata of physical activity on BMI in control (N = 6784) participants from PROMIS. (DOCX 53 kb) [file 12881_2015_259_MOESM4_ESM.docx]

**Table S3** Association of individual 95 SNPs with different strata of physical activity on BMI in control (N= 6,784) participants from PROMIS.

|  | | **Inactive (N= 2,749)** | | | **Moderate(N=3,401)** | | | **Active (N= 634)** | | |  |
| --- | --- | --- | --- | --- | --- | --- | --- | --- | --- | --- | --- |
| **SNP** | **Nearest Gene** | β (kg/m2) | SE | *P-value* | β (kg/m^2^) | SE | *P-value* | β (kg/m^2^) | SE | *P-value* | *P*_interaction_ |
| rs1000940 | *RABEP1* | -0.17 | 0.11 | 0.14 | 0.13 | 0.10 | 0.19 | -0.08 | 0.24 | 0.75 | 0.24 |
| rs10132280 | *STXBP6* | 0.04 | 0.14 | 0.77 | -0.09 | 0.12 | 0.48 | -0.06 | 0.28 | 0.83 | 0.57 |
| rs1016287 | *FLJ30838* | 0.17 | 0.13 | 0.18 | 0.28 | 0.11 | 0.01 | -0.14 | 0.26 | 0.58 | 0.57 |
| rs10182181 | *ADCY3* | 0.24 | 0.11 | 0.03 | 0.04 | 0.10 | 0.67 | -0.10 | 0.23 | 0.65 | 0.11 |
| rs10733682 | *LMX1B* | -0.06 | 0.11 | 0.61 | -0.12 | 0.10 | 0.24 | -0.08 | 0.23 | 0.72 | 0.68 |
| rs10938397 | *GNPDA2* | 0.07 | 0.11 | 0.54 | 0.05 | 0.10 | 0.61 | 0.23 | 0.22 | 0.31 | 0.91 |
| rs10968576 | *LINGO2* | 0.05 | 0.14 | 0.74 | 0.03 | 0.12 | 0.82 | 0.93 | 0.29 | 0.00 | 0.10 |
| rs11030104 | *BDNF* | 0.06 | 0.13 | 0.63 | 0.21 | 0.11 | 0.07 | 0.40 | 0.26 | 0.12 | 0.25 |
| rs11057405 | *CLIP1* | -0.75 | 0.36 | 0.04 | -0.13 | 0.32 | 0.69 | 1.19 | 0.79 | 0.13 | 0.014 |
| rs11126666 | *KCNK3* | 0.09 | 0.12 | 0.44 | 0.02 | 0.11 | 0.87 | -0.06 | 0.26 | 0.82 | 0.77 |
| rs11165643 | *PTBP2* | 0.17 | 0.11 | 0.12 | 0.16 | 0.10 | 0.10 | 0.58 | 0.23 | 0.01 | 0.26 |
| rs11191560 | *NT5C2* | 0.04 | 0.14 | 0.78 | 0.01 | 0.12 | 0.96 | 0.31 | 0.30 | 0.29 | 0.53 |
| rs11583200 | *ELAVL4* | 0.00 | 0.11 | 0.98 | 0.01 | 0.10 | 0.95 | 0.27 | 0.22 | 0.23 | 0.43 |
| rs1167827 | *HIP1* | 0.05 | 0.11 | 0.66 | 0.04 | 0.10 | 0.71 | 0.18 | 0.23 | 0.43 | 0.98 |
| rs11688816 | *EHBP1* | -0.06 | 0.11 | 0.56 | -0.12 | 0.10 | 0.23 | -0.14 | 0.23 | 0.57 | 0.96 |
| rs11727676 | *HHIP* | 0.00 | 0.38 | 0.99 | 0.01 | 0.35 | 0.98 | 0.69 | 0.72 | 0.34 | 0.57 |
| rs11847697 | *PRKD1* | 0.42 | 0.22 | 0.05 | 0.26 | 0.19 | 0.15 | 0.21 | 0.47 | 0.66 | 0.50 |
| rs12286929 | *CADM1* | 0.05 | 0.11 | 0.66 | 0.06 | 0.10 | 0.57 | -0.06 | 0.24 | 0.79 | 0.78 |
| rs12401738 | *FUBP1* | -0.10 | 0.14 | 0.47 | -0.04 | 0.12 | 0.76 | 0.10 | 0.29 | 0.73 | 0.43 |
| rs12429545 | *OLFM4* | 0.02 | 0.15 | 0.88 | 0.07 | 0.13 | 0.60 | 0.04 | 0.29 | 0.88 | 0.98 |
| rs12446632 | *GPRC5B* | 0.36 | 0.28 | 0.19 | -0.10 | 0.23 | 0.67 | 0.02 | 0.54 | 0.97 | 0.32 |
| rs12566985 | *FPGT-TNNI3K* | 0.00 | 0.11 | 0.97 | 0.00 | 0.10 | 0.97 | -0.01 | 0.24 | 0.96 | 0.98 |
| rs12885454 | *PRKD1* | -0.16 | 0.12 | 0.17 | 0.12 | 0.10 | 0.24 | 0.11 | 0.23 | 0.63 | 0.11 |
| rs12940622 | *RPTOR* | 0.02 | 0.12 | 0.84 | 0.12 | 0.10 | 0.25 | -0.36 | 0.25 | 0.15 | 0.64 |
| rs13021737 | *TMEM18* | 0.40 | 0.14 | 0.01 | 0.17 | 0.13 | 0.20 | -0.07 | 0.30 | 0.80 | 0.10 |
| rs13078960 | *CADM2* | -0.14 | 0.17 | 0.41 | 0.19 | 0.16 | 0.22 | 0.59 | 0.37 | 0.11 | 0.037 |
| rs13191362 | *PARK2* | 0.01 | 0.18 | 0.96 | -0.02 | 0.17 | 0.91 | 0.07 | 0.37 | 0.85 | 0.76 |
| rs13201877 | *IFNGR1* | 0.13 | 0.21 | 0.54 | 0.19 | 0.18 | 0.28 | 0.36 | 0.41 | 0.39 | 0.66 |
| rs1441264 | *MIR548A2* | 0.24 | 0.12 | 0.05 | 0.00 | 0.11 | 1.00 | 0.19 | 0.25 | 0.45 | 0.27 |
| rs1460676 | *FIGN* | 0.02 | 0.16 | 0.92 | -0.24 | 0.14 | 0.08 | 0.21 | 0.33 | 0.51 | 0.90 |
| rs1516725 | *ETV5* | 0.25 | 0.15 | 0.09 | 0.16 | 0.13 | 0.21 | 0.56 | 0.32 | 0.08 | 0.83 |
| rs1528435 | *UBE2E3* | 0.06 | 0.12 | 0.59 | -0.03 | 0.10 | 0.77 | 0.08 | 0.24 | 0.73 | 0.68 |
| rs1558902 | *FTO* | 0.32 | 0.12 | 0.01 | 0.24 | 0.10 | 0.02 | 0.43 | 0.24 | 0.08 | 0.96 |
| rs16851483 | *RASA2* | 0.26 | 0.18 | 0.14 | 0.25 | 0.16 | 0.11 | 0.13 | 0.39 | 0.73 | 0.81 |
| rs16907751 | *ZBTB10* | -0.01 | 0.14 | 0.96 | -0.04 | 0.13 | 0.74 | 0.32 | 0.29 | 0.27 | 0.67 |
| rs16951275 | *MAP2K5* | 0.09 | 0.12 | 0.42 | 0.08 | 0.10 | 0.44 | 0.07 | 0.25 | 0.77 | 0.94 |
| rs17001654 | *SCARB2* | 0.14 | 0.19 | 0.46 | 0.24 | 0.17 | 0.16 | -0.43 | 0.41 | 0.29 | 0.37 |
| rs17094222 | *HIF1AN* | -0.12 | 0.16 | 0.45 | 0.22 | 0.14 | 0.12 | 0.31 | 0.33 | 0.35 | 0.07 |
| rs17203016 | *CREB1* | 0.17 | 0.16 | 0.28 | -0.14 | 0.13 | 0.29 | 0.26 | 0.30 | 0.38 | 0.50 |
| rs17405819 | *HNF4G* | -0.02 | 0.12 | 0.85 | 0.10 | 0.11 | 0.36 | -0.19 | 0.25 | 0.44 | 1.00 |
| rs17724992 | *PGPEP1* | -0.09 | 0.11 | 0.45 | -0.15 | 0.10 | 0.13 | -0.25 | 0.22 | 0.27 | 0.50 |
| rs1808579 | *C18orf8* | -0.14 | 0.11 | 0.21 | -0.14 | 0.10 | 0.15 | -0.02 | 0.23 | 0.92 | 0.78 |
| rs1885988 | MTIF3 | 0.05 | 0.19 | 0.80 | -0.04 | 0.17 | 0.82 | 0.80 | 0.45 | 0.08 | 0.39 |
| rs1928295 | *TLR4* | 0.13 | 0.11 | 0.25 | -0.05 | 0.10 | 0.62 | 0.17 | 0.24 | 0.49 | 0.61 |
| rs2033529 | *TDRG1* | 0.30 | 0.14 | 0.03 | -0.06 | 0.13 | 0.62 | 0.22 | 0.29 | 0.45 | 0.19 |
| rs2033732 | *RALYL* | 0.10 | 0.13 | 0.44 | 0.06 | 0.11 | 0.60 | 0.28 | 0.27 | 0.29 | 0.85 |
| rs205262 | *C6orf106* | -0.14 | 0.14 | 0.29 | -0.01 | 0.12 | 0.91 | -0.19 | 0.28 | 0.51 | 0.73 |
| rs2075650 | *TOMM40* | 0.29 | 0.18 | 0.10 | -0.17 | 0.16 | 0.28 | 0.45 | 0.37 | 0.23 | 0.60 |
| rs2080454 | *CBLN1* | 0.10 | 0.13 | 0.42 | 0.17 | 0.11 | 0.12 | -0.45 | 0.26 | 0.08 | 0.26 |
| rs2112347 | *POC5* | -0.13 | 0.11 | 0.24 | 0.10 | 0.10 | 0.29 | 0.04 | 0.22 | 0.84 | 0.18 |
| rs2121279 | *LRP1B* | 0.37 | 0.24 | 0.12 | 0.16 | 0.23 | 0.49 | 0.19 | 0.50 | 0.70 | 0.37 |
| rs2176040 | *LOC646736* | 0.20 | 0.13 | 0.12 | 0.03 | 0.11 | 0.81 | -0.03 | 0.26 | 0.91 | 0.15 |
| rs2176598 | *HSD17B12* | 0.23 | 0.14 | 0.11 | -0.19 | 0.13 | 0.14 | -0.05 | 0.30 | 0.86 | 0.07 |
| rs2207139 | *TFAP2B* | 0.31 | 0.14 | 0.02 | 0.20 | 0.12 | 0.09 | -0.03 | 0.27 | 0.92 | 0.25 |
| rs2245368 | *PMS2L11* | -0.07 | 0.12 | 0.53 | 0.12 | 0.10 | 0.23 | 0.24 | 0.24 | 0.32 | 0.12 |
| rs2287019 | *QPCTL* | 0.14 | 0.15 | 0.35 | -0.01 | 0.13 | 0.96 | 0.49 | 0.33 | 0.14 | 0.90 |
| rs2365389 | *FHIT* | 0.10 | 0.11 | 0.35 | -0.08 | 0.10 | 0.40 | 0.05 | 0.22 | 0.83 | 0.48 |
| rs2650492 | *SBK1* | -0.01 | 0.14 | 0.94 | -0.07 | 0.13 | 0.59 | -0.24 | 0.31 | 0.44 | 0.63 |
| rs2820292 | *NAV1* | 0.05 | 0.11 | 0.66 | -0.03 | 0.10 | 0.80 | 0.12 | 0.23 | 0.60 | 0.93 |
| rs2836754 | *ETS2* | 0.35 | 0.12 | 0.00 | 0.16 | 0.11 | 0.14 | 0.30 | 0.24 | 0.20 | 0.54 |
| rs29941 | *KCTD15* | 0.14 | 0.12 | 0.23 | -0.04 | 0.10 | 0.70 | 0.31 | 0.23 | 0.18 | 0.83 |
| rs3101336 | *NEGR1* | 0.17 | 0.12 | 0.15 | 0.12 | 0.10 | 0.24 | 0.07 | 0.24 | 0.77 | 0.65 |
| rs3736485 | *DMXL2* | 0.02 | 0.11 | 0.89 | 0.12 | 0.10 | 0.23 | -0.06 | 0.23 | 0.80 | 0.91 |
| rs3810291 | *ZC3H4* | 0.06 | 0.11 | 0.59 | 0.14 | 0.10 | 0.17 | -0.22 | 0.22 | 0.32 | 0.63 |
| rs3817334 | *MTCH2* | 0.12 | 0.12 | 0.31 | -0.02 | 0.11 | 0.83 | 0.21 | 0.25 | 0.41 | 0.99 |
| rs3849570 | *GBE1* | 0.01 | 0.12 | 0.93 | 0.16 | 0.10 | 0.12 | -0.35 | 0.24 | 0.15 | 0.68 |
| rs3888190 | *ATP2A1(SH2B1)* | 0.10 | 0.13 | 0.42 | 0.07 | 0.11 | 0.57 | -0.07 | 0.27 | 0.79 | 0.70 |
| rs4256980 | *TRIM66* | 0.18 | 0.11 | 0.11 | -0.05 | 0.10 | 0.59 | 0.38 | 0.23 | 0.10 | 0.53 |
| rs4740619 | *C9orf93* | -0.17 | 0.11 | 0.12 | -0.21 | 0.10 | 0.03 | -0.32 | 0.23 | 0.16 | 0.70 |
| rs4787491 | *INO80E* | 0.07 | 0.11 | 0.51 | -0.04 | 0.10 | 0.70 | -0.35 | 0.23 | 0.13 | 0.09 |
| rs492400 | *USP37* | -0.07 | 0.12 | 0.58 | 0.02 | 0.10 | 0.88 | 0.03 | 0.25 | 0.91 | 0.80 |
| rs543874 | *SEC16B* | 0.32 | 0.15 | 0.04 | 0.22 | 0.14 | 0.11 | -0.59 | 0.33 | 0.07 | 0.06 |
| rs6091540 | *ZFP64* | 0.10 | 0.12 | 0.41 | 0.10 | 0.11 | 0.36 | -0.32 | 0.25 | 0.20 | 0.33 |
| rs6465468 | *ASB4* | -0.42 | 0.15 | 0.00 | -0.22 | 0.12 | 0.07 | 0.11 | 0.30 | 0.72 | 0.10 |
| rs6477694 | *EPB41L4B* | 0.23 | 0.11 | 0.04 | 0.04 | 0.10 | 0.68 | -0.10 | 0.23 | 0.68 | 0.16 |
| rs6567160 | *MC4R* | 0.41 | 0.12 | 0.00 | 0.23 | 0.10 | 0.03 | 0.20 | 0.24 | 0.41 | 0.30 |
| rs657452 | *AGBL4* | 0.00 | 0.11 | 0.97 | -0.08 | 0.10 | 0.44 | 0.03 | 0.23 | 0.90 | 0.96 |
| rs6804842 | *RARB* | 0.06 | 0.11 | 0.58 | -0.01 | 0.10 | 0.95 | 0.21 | 0.22 | 0.35 | 0.97 |
| rs7138803 | *BCDIN3D (FAIM2)* | 0.12 | 0.11 | 0.29 | 0.01 | 0.10 | 0.90 | 0.01 | 0.23 | 0.96 | 0.44 |
| rs7141420 | *NRXN3* | -0.09 | 0.11 | 0.43 | 0.03 | 0.10 | 0.79 | -0.39 | 0.24 | 0.11 | 0.99 |
| rs7164727 | *LOC100287559* | 0.14 | 0.11 | 0.21 | 0.19 | 0.10 | 0.06 | -0.41 | 0.24 | 0.08 | 0.28 |
| rs7239883 | *LOC284260* | -0.05 | 0.12 | 0.66 | 0.13 | 0.11 | 0.22 | 0.44 | 0.25 | 0.08 | 0.07 |
| rs7243357 | *GRP* | 0.00 | 0.13 | 1.00 | 0.21 | 0.12 | 0.07 | 0.08 | 0.27 | 0.78 | 0.45 |
| rs758747 | *NLRC3* | 0.14 | 0.12 | 0.23 | 0.19 | 0.11 | 0.08 | 0.48 | 0.25 | 0.05 | 0.27 |
| rs7599312 | *ERBB4* | -0.02 | 0.14 | 0.89 | 0.02 | 0.13 | 0.87 | 0.32 | 0.31 | 0.29 | 0.43 |
| rs7715256 | *GALNT10* | 0.03 | 0.11 | 0.80 | -0.22 | 0.10 | 0.02 | -0.36 | 0.23 | 0.12 | 0.048 |
| rs7899106 | *GRID1* | 0.44 | 0.35 | 0.21 | 0.65 | 0.30 | 0.03 | 0.05 | 0.73 | 0.95 | 0.88 |
| rs7903146 | *TCF7L2* | 0.17 | 0.12 | 0.15 | 0.34 | 0.10 | 0.00 | 0.00 | 0.25 | 0.99 | 0.98 |
| rs9374842 | *LOC285762* | 0.04 | 0.12 | 0.76 | -0.06 | 0.10 | 0.59 | -0.08 | 0.24 | 0.73 | 0.80 |
| rs9400239 | *FOXO3* | -0.02 | 0.11 | 0.88 | 0.08 | 0.10 | 0.43 | 0.00 | 0.23 | 1.00 | 0.89 |
| rs9540493 | *MIR548X2* | 0.05 | 0.11 | 0.65 | -0.05 | 0.10 | 0.60 | 0.23 | 0.24 | 0.33 | 0.86 |
| rs9641123 | *CALCR* | 0.35 | 0.11 | 0.00 | 0.10 | 0.10 | 0.30 | 0.38 | 0.23 | 0.10 | 0.31 |
| rs977747 | *TAL1* | -0.18 | 0.11 | 0.10 | -0.01 | 0.10 | 0.92 | -0.04 | 0.23 | 0.85 | 0.24 |
| rs9914578 | *SMG6* | -0.09 | 0.12 | 0.44 | -0.17 | 0.10 | 0.10 | -0.17 | 0.24 | 0.50 | 0.74 |
| rs9925964 | *KAT8* | -0.14 | 0.14 | 0.31 | -0.02 | 0.12 | 0.85 | 0.25 | 0.29 | 0.39 | 0.17 |

Analyses were adjusted for: age, age^2^, sex and genetic principal components.
